# Method for Training Convolutional Neural Networks for In Situ Plankton Image Recognition and Classification Based on the Mechanisms of the Human Eye

**DOI:** 10.3390/s20092592

**Published:** 2020-05-02

**Authors:** Xuemin Cheng, Yong Ren, Kaichang Cheng, Jie Cao, Qun Hao

**Affiliations:** 1 Shenzhen International Graduate School, Tsinghua University, Shenzhen 518055, China; ry17@mails.tsinghua.edu.cn (Y.R.); ckc17@mails.tsinghua.edu.cn (K.C.); 2 School of Optics and Photonics, Beijing Institute of Technology, Beijing 100081, China; caojie@bit.edu.cn (J.C.); qhao@bit.edu.cn (Q.H.)

**Keywords:** cartesian and polar coordinate, classification and recognition, two features combination, mechanisms of human eye, convolutional neural network

## Abstract

In this study, we propose a method for training convolutional neural networks to make them identify and classify images with higher classification accuracy. By combining the Cartesian and polar coordinate systems when describing the images, the method of recognition and classification for plankton images is discussed. The optimized classification and recognition networks are constructed. They are available for in situ plankton images, exploiting the advantages of both coordinate systems in the network training process. Fusing the two types of vectors and using them as the input for conventional machine learning models for classification, support vector machines (SVMs) are selected as the classifiers to combine these two features of vectors, coming from different image coordinate descriptions. The accuracy of the proposed model was markedly higher than those of the initial classical convolutional neural networks when using the in situ plankton image data, with the increases in classification accuracy and recall rate being 5.3% and 5.1% respectively. In addition, the proposed training method can improve the classification performance considerably when used on the public CIFAR-10 dataset.

## 1. Introduction

Plankton, the tiny oceanic organisms in the marine realm, play a critical role in marine research and they are highly influenced by their environmental conditions [1,2]. Plankton can be observed as recorded images that are captured by underwater imaging systems [3]. As far as we know, automatic and accurate identification of these tiny organisms is essential for real-time monitoring of the marine ecology as well as for advanced assessment of water quality and the marine environment [4].

To perform ecological monitoring using an imaging system, an image acquisition technique must be applied. However, owing to the presence of organic matter and suspended particles, underwater visibility is highly limited. The ideal visibility, that is, the visibility in the case of clear seawater, is typically approximately 20 m. However, the visibility under the condition of in situ imaging, may be only a few meters in turbid seawater [5]. It is limited because of the attenuation of light as it propagates through the seawater [6]. Light attenuation blurs the background of the captured image, and the living organisms and suspended matter found in complex underwater environments may cause noise pollution and interference [7]. Light from artificial sources used to improve visibility also undergoes scattering, which adversely affects the image quality [8]. These in situ imaging conditions lead to a deterioration of the image, and the plankton features become very tiny.

**Figure 1:**
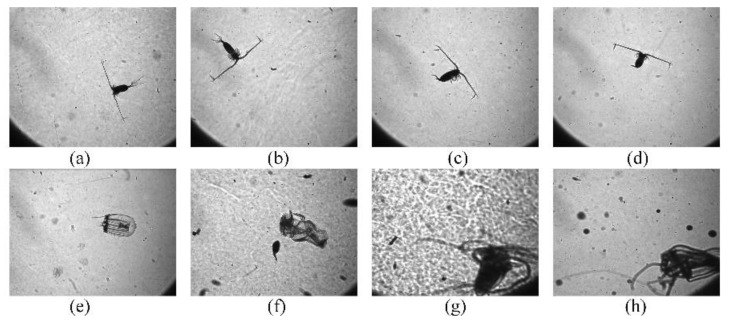
(**a**–**d**): Copepods of different positions and attitudes; (**e**–**h**): jellyfishes of different positions and attitudes.

Furthermore, the plankton images are required to be classified accurately for further research once the underwater images have been acquired. Some early methods are mainly based on morphology and texture [9]. Then, manual design feature descriptors are proposed to extract image features, and then these features are used for classification by using methods such as support vector machines or “random forests” [10,11]. Traditional classification uses low-level manual design functions, but these traditional methods often have poor generalization capabilities and are difficult to be used in new imaging situations. Then deep learning is proposed, possessing the characteristics of automatic feature learning and feature extraction, its generalization performance is better [12]. The latest trends in plankton classification are also based on deep learning and convolutional neural network [13]. The convolutional neural network models proposed recently are as follows. LeCun et al. introduced the first convolutional neural network model (LeNet), with a rapid growth [14,15], however, LeNet is adept in handling simple images, e.g., handwriting recognition. It has weak generalization ability and is not applicable for tiny feature in the images. In 2012, the AlexNet model was proposed [16]. In this model, the network convergence rate is higher, and over-fitting is effectively prevented [14]. At that time, convolutional neural networks had been developed to be applicable for large-scale complex images with an increasing size [17]. VGGNet was proposed in 2014. This may be considered as an upgraded version of AlexNet which is a convolutional neural network with even more layers and greater depth [18]. More importantly, the VGGNet showed that the accuracy of image recognition can be improved by increasing the depth of the convolutional network. It also revealed that multiple consecutive small-scale convolutions contain more nonlinear expressions than a single large-scale convolution, and the smaller-sized convolution kernels can reduce the computation cost and hasten convergence [14]. In the GoogLeNet model, the inception structure was used to replace the convolution and the activation functions [19]. In this model, the kernel size is not chosen based on human experience; instead, the complexity of the network is increased by increasing its width. The program adaptively learns how to choose the size of the convolution kernel [14]. In addition, a unique feature of the GoogLeNet model is that it improves the generalization capability of the convolution model and increases the complexity of the network structure, using a 1 × 1 convolution operation while maintaining the order of magnitude of the number of parameters [14]. Although increasing network depth can improve recognition accuracy, it also diminishes the gradient and degrades the depth of the network [14]. To solve this problem, He et al. introduced a residual block in the neural network. The resulting model, ResNet, is a convolutional neural network with hundreds of parameter layers, and its residual structures are constructed by introducing shortcuts between the parameter layers [20]. These changes increase the convergence rate and also improve recognition accuracy [14]. As stated above, GoogLeNet solves the width extension problem of convolutional neural networks while ResNet solves the deep extension problem. In contrast, DenseNet is a new type of convolutional neural network model [21]. DenseNet fully exploits the characteristic information hidden in the front layer of the network, starting from the feature level [14]. In other words, starting from the feature level, DenseNet establishes the connections between the different layers. This approach alleviates the problem of decrease in the gradient with the increasing depth of higher memory requirements for model training. Its powerful learning and convergence abilities are being exploited for feature extraction and classification. However, these models might not yet be used directly in an efficient way to process, extract, and classify in situ plankton images, which are imaged in turbid water as discussed above. This is because the convolutional neural network model is based on the image convolution operations, and the existing convolution module is generally and translationally invariant [22]. However, if the target is rotated by a certain angle, it will not be recognized by the convolution neural network. Thus, this will affect the generalization capability of the network as well as its classification accuracy. Moreover, for in situ plankton images as shown in Figure 1, it is very common for the same creature to appear possessing different postures and positions.

To address this problem, we propose a new method for image classification and network training, combining the translational and rotational features, paying attention to the in situ plankton image. Polar coordinate representation based on the mechanisms of the human eye is used to describe the rotational features of the target, and it is used for the transformation into translational ones for the input of the neural network. During the learning process, the rotational features of the target are learned indirectly, and they are expected to improve the generalization capability and classification accuracy of the network. In the proposed method, the features of the target before and after rotation are extracted and combined to improve the classification accuracy and recall rate. Using this method, we achieve satisfactory results for the Bering Sea plankton image dataset (acquired and constructed in the laboratory [23]) and the CIFAR10 dataset [24].

The rest of this paper is structured as follows: Section 2 discusses the proposed methods, including those used for problem analysis, model building, and finding the solution; Section 3 describes the proposed training method which combines a neural network with the polar mechanisms similar to what are used in the human eye, as well as the results of the tests conducted to evaluate the performance of the proposed model; and Section 4 summarizes the primary conclusions of the study and the direction that future work on the topic should take.

## 2. Materials and Methods

### 2.1. Existing Method

**Figure 2:**
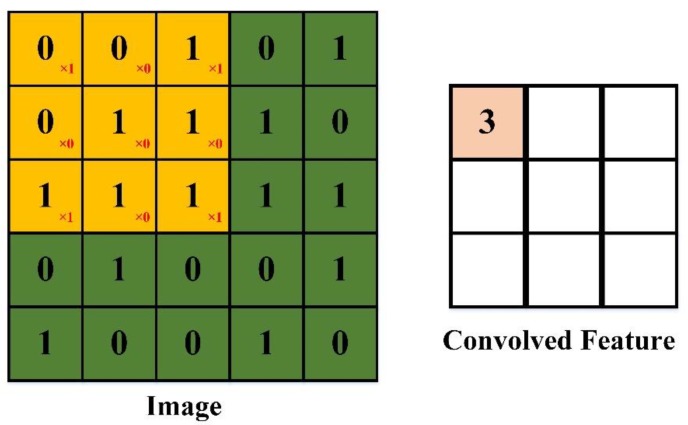
Schematic illustration of convolutional neural networks.

In Euclidean geometry, translation is a geometric transformation that moves every point on an image or in a given space by the same distance and in the same direction. For example, in an image classification task, the target should remain the same (i.e., have the same tag) irrespective of where it moves to in the image. However, it is difficult to remain translationally invariant when handling image processing and classification [22]. Convolutional neural networks can partially solve the problem of translation invariance by introducing local connections and weight sharing [22]. The convolution process is defined as feature detection at a specific location [14]. This implies that, regardless of where the target appears in the image, the system should detect the same features and output the same response. If the target is moved to a different position in the image, the convolution kernel will not detect the features of the target until the kernel moves to a new target location [22], and the convolution kernel is invariant during the moving process. This function of the kernel together with that of the largest pooling layer ensures the translational invariance of the target in the convolutional neural network, as demonstrated in Figure 2.

However, the previous research has showed that the convolution process is susceptible to the rotation of the image [22]. In this manner, the neural network will be made to partially learn features when considering the rotated images. Thus, it is necessary to use and accumulate a large amount of target data to perform rotation and translation enhancements on these sample data, and use more datasets for network training [17,25]. Furthermore, this requirement is often difficult to satisfy when working with a small scale of the dataset (e.g., the in situ images) similar to this study in which in situ-obtained marine plankton images are used. This is because the images must be acquired in the field, and extensive testing in the ocean will be required to obtain a sufficiently large dataset. Hence, it is essential to improve the classification accuracy, using smaller datasets.

### 2.2. The Proposed Method

**Figure 3:**
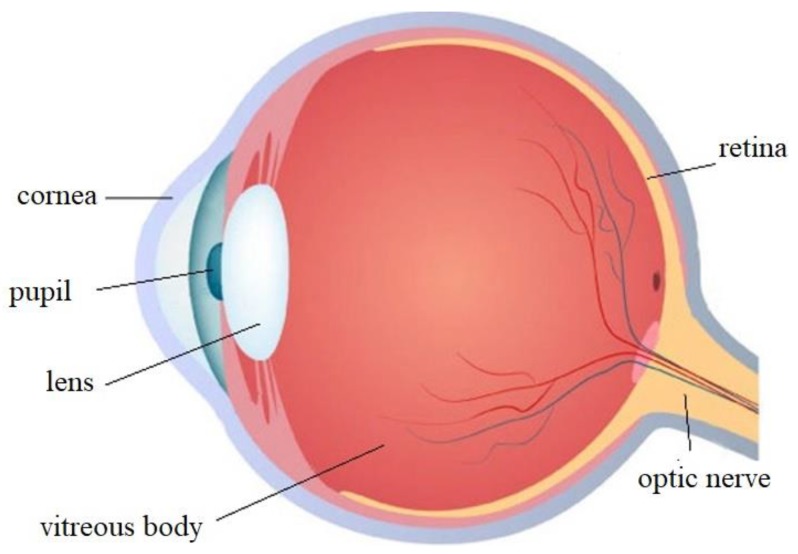
Structure of the eyeball.

To solve the problem of the rotational invariance of image features, we sought to mimic the imaging mechanisms of the human eye. Figure 3 shows the structure of an eyeball [26]. Studies have shown that external light first enters the eyeball through the cornea at the pupil. It then passes through the lens and vitreous body to reach the retina [27]. In the retina, the light signal is converted into an electrical one, and it is subjected to initial processing [27,28]. The process of recognizing, perceiving, and understanding the external signals are completed in the visual cortex. The retinal imaging mechanism of the human eye is an uniformed sampling process which can be best described by transformation in a logarithmic polar coordinate [29]. The logarithmic polar coordinate system can transform an image in a Cartesian coordinate system into one in a logarithmic polar coordinate system based on a set of transformation laws [30].

(1) $$\begin{array}{l} \rho =\log_{a}\sqrt{{\left(x-x_{c}\right)}^{2}+{\left(y-y_{c}\right)}^{2}}\\ \theta =\arctan(\frac{y-y_{c}}{x-x_{c}}) \end{array}$$

Considering the logarithmic polar coordinate point (ρ,θ), where a represents the base number (typically 10 or e), (x,y) represents the point of Cartesian coordinate, ρ denotes the logarithmic radial distance from the center point $\left(x_{c},y_{c}\right)$, and θ denotes an angle. However, the log polar coordinate systems tend to display mainly the central section of an image. If point (x,y) is far from the center point $\left(x_{c},y_{c}\right)$, Δρ, the increment of ρ, varies very slowly and nonlinearly. Given that we wished to obtain rotationally invariant images that retained the image features, we opted to describe the images, using a polar coordinate system:

(2) $$\begin{array}{l} \rho =\sqrt{{\left(x-x_{c}\right)}^{2}+{\left(y-y_{c}\right)}^{2}}\\ \theta =\arctan(\frac{y-y_{c}}{x-x_{c}}) \end{array}$$

where ρ denotes radial distance from the center $\left(x_{c},y_{c}\right)$.

**Figure 4:**
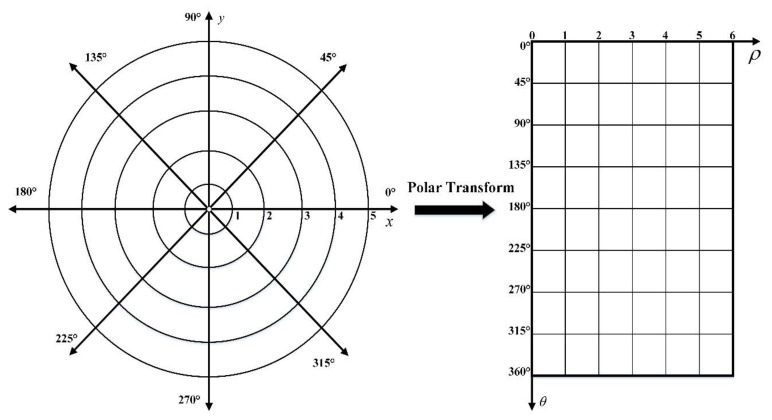
Correspondence of (**left**) Cartesian coordinates and (**right**) polar coordinates.

**Figure 5:**
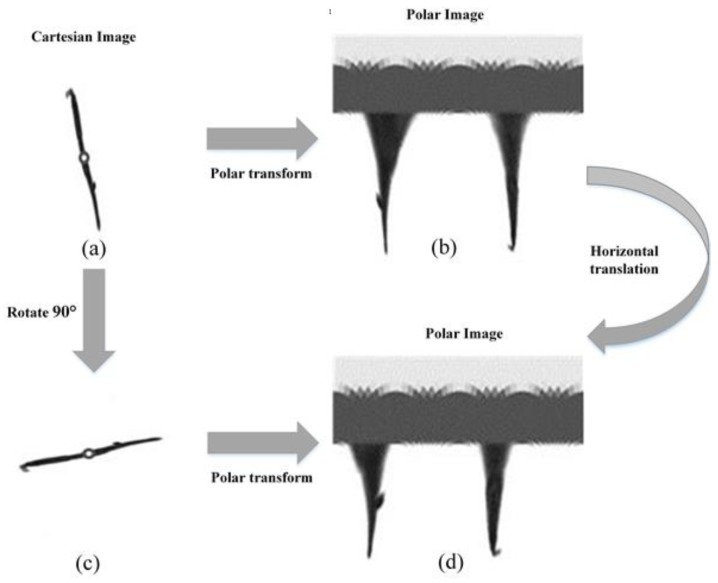
Description of rotational invariance. The rotation of an object can be illustrated as a type of horizontal translation. (**a**) an arrow worm (kind of plankton) image described by Cartesian coordinates; this is common and ordinary.; (**b**) description by polar coordinates; (**c**) arrow worm image in (**a**) was rotated 90° clockwise; (**d**) description by polar coordinates.

Assuming ρ and θ lie along the vertical and horizontal axes respectively, we obtain the coordinate system shown in Figure 4. Note that the origin in both coordinate systems is taken to be in the upper left corner. The benefit of this new coordinate space is that a simple scale and a rotation change may be induced by directly modifying the (ρ,θ) data [31]. If the images in the Cartesian coordinates system rotate ϕ degrees, they will translate ϕ horizontally in the polar coordinate. Likewise, the scaling can also be used for vertical translation as shown in Figure 5.

Considering that feature learning and extraction in convolutional neural networks had worked well [14], the convolutional neural networks were applied to extract feature vectors in images and to describe them by both Cartesian coordinates and polar coordinates. Fusing the two types of vectors and using them as inputs for the conventional machine learning models for classification, another classifier to combine them is needed in order to describe and integrate these two features of vectors that come from different image coordinates. Support vector machines (SVMs) were selected as the classifiers as they had been proven to have properly performed the two-class and multiclass classification tasks [23,32], and had been used successfully in the classification of plankton images [33]. It is assumed that SVMs can find the optimal hyperplane in the feature space. This results in the greatest separation between the positive and negative samples in the training set [34]. In addition, a certain amount of fault tolerance can be retained when using the concept of soft interval in order to improve the robustness and classification accuracy of SVMs. Considering that there will exist morphological differences between conspecific plankton populations from different marine regions, the classification should be performed, using the model of higher fault-tolerance [35]. Thus, a multiclass SVM model developed by our research group is applied using this method [36]. The SVM classifier is used to classify the dataset based on a combination of two kinds of features that are described in Cartesian coordinate and polar coordinate systems.

For features in Cartesian coordinate and polar coordinate systems, we separately input the Cartesian coordinate images as $I_{c}$ and polar coordinate images as $I_{p}$ into two different convolutional neural networks with the same structure and different training parameters. The two networks are trained separately without interference. As the fully connected layer can describe the features learned by the entire network model, we use the output of the fully connected layer of the classical convolutional neural network as the features’ eigenvector ($V_{c}$) obtained from Image $I_{c}$, and the features of eigenvector ($V_{p}$) was obtained from Image $I_{p}$. By splicing the features, we obtain the vector eigenvector,$V=\left[\begin{array}{c} V_{c}\\ V_{p} \end{array}\right]$, which has both the Cartesian coordinate features and the polar coordinate features.

**Figure 6:**
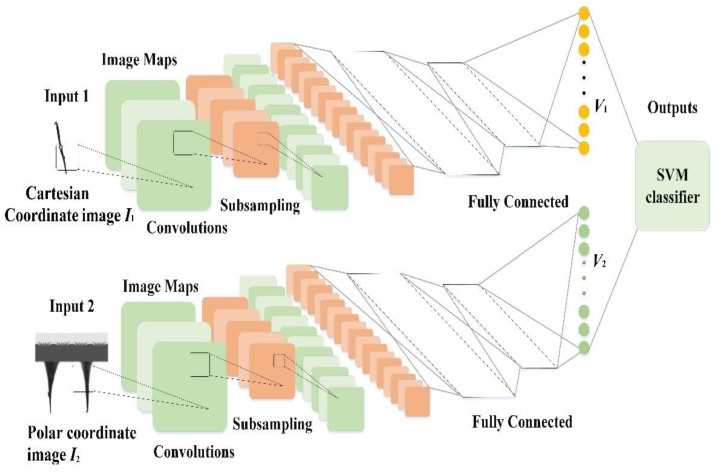
Structure of the proposed model. Note: SVM = support vector machine.

**Figure 7:**
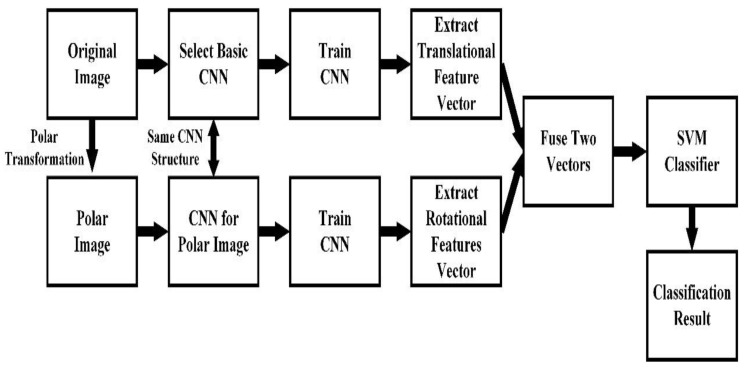
Block diagram of the proposed method. Note: CNN= convolutional neural network.

**Figure 8:**
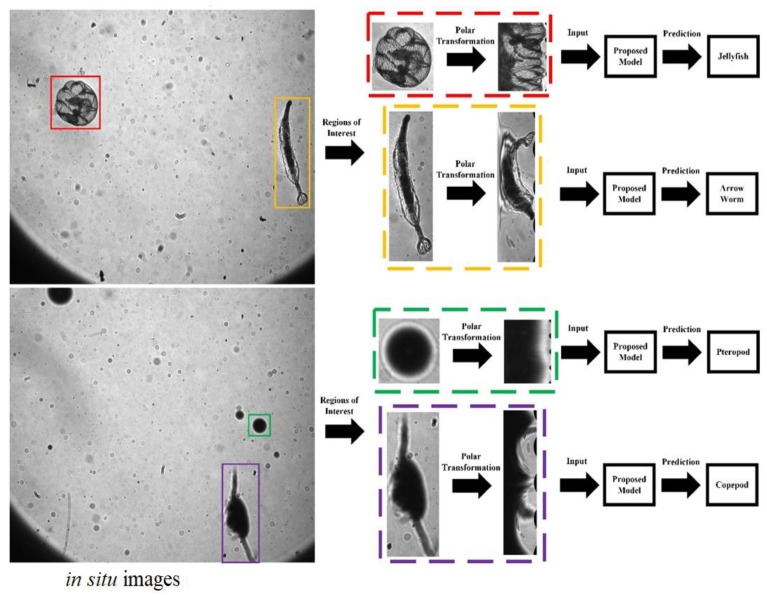
Application of the proposed method.

As shown in Figure 6, after the feature extraction process, the eigenvector (V) is inputted into the multiclass SVM to realize a global optimization. In this case, with two different kinds of features contained, the parameters of the SVM classifier are optimized separately. The process of the proposed research is shown in Figure 7. To illustrate the application of the proposed method, the sample in situ images captured by the undersea imaging device, and the process of region of interest (ROI) extraction and classification are also shown in Figure 8.

## 3. Experimental Design and Analysis

### 3.1. Dataset Used

We used two datasets: the in situ plankton dataset and the CIFAR-10 dataset in order to further discuss the robustness of the algorithm. The images in the plankton dataset were acquired in the Bering Sea, using PlanktonScope, an in situ underwater imager [37]. The plankton images in the datasets used for training and testing were extracted from the original image dataset, using an ROI extraction program [38]. The samples in the training dataset included those corresponding to six planktonic taxa (arrow worm, copepod, fish larvae, jellyfish, krill, and pteropod) as well as the negative samples. After obtaining the initial ROI samples, the sampling scale was increased, using the mirroring and rotation operations. The plankton datasets comprised seven classes. The training set comprised 2048 examples from each category while the testing set comprised 512 examples from each category. Altogether, the total number of the samples across all seven training sets was 14,336 while the total number of samples across all seven validation sets was 3584.

For generalization, the CIFAR-10 dataset was used to measure the performance of the convolutional neural networks [24]. This dataset comprised 60,000 color images which were divided into 10 categories of 6000 images each. For this dataset, we used 50,000 images for training and 10,000 images for testing.

### 3.2. Experimental Procedure

To evaluate the classification accuracy and the efficiency of the human-eye-based neural network training method proposed in this study, we performed several sets of comparative tests (Table 1 and Table 2 were performed, using MATLAB 2018b on a Core i3 7100 CPU with 32 GB RAM running the Ubuntu operating system (16.04)). The fully connected layer is the structural layer of the neural network which can comprehensively describe the characteristics of the network samples. However, there may exist multiple types of fully connected layers in the actual network structure [14]. Therefore, to ensure a better classification accuracy, we consistently used the output of the first fully connected layer of the classical convolutional neural network as the input for the multiclass SVM model.

To verify the effect of the proposed model in extracting rotation features, we also had the original image rotated with a step of 30 degrees, flipped up and down, and flipped left and right respectively, and called as the augmented dataset for data augmentation, which is 14 times the size of the original. Thereafter, a series of comparative tests were performed to calculate their classification accuracy, recall rate, and run time of each model, using the in situ plankton image dataset (Table 1). In Models 1–5, we fine-tuned the classical convolutional neural network, using the training set, and then determined the classification accuracy and run time, using the testing set. In Models 6–10, we fine-tuned the same classical convolutional neural network corresponding to Models 1–5, using the augmented training set. In Models 11–15, we extracted feature maps from the corresponding Models (1–5) after the completion of the first fully connected layer. Thereafter, we trained the multiclass SVM model, using the training set and determined the classification accuracy and run time, using the testing set. In Models 16–20, we combined the features extracted from the dataset with the corresponding Models (11–15) after performing polar coordinate transformation. We then calculated the classification accuracy and run time, using the testing set. To validate the performance of each algorithm, we also performed the tests, using the CIFAR-10 dataset (Table 2) in the same procedure.

### 3.3. Analysis of Evaluation Results

**Figure 9:**
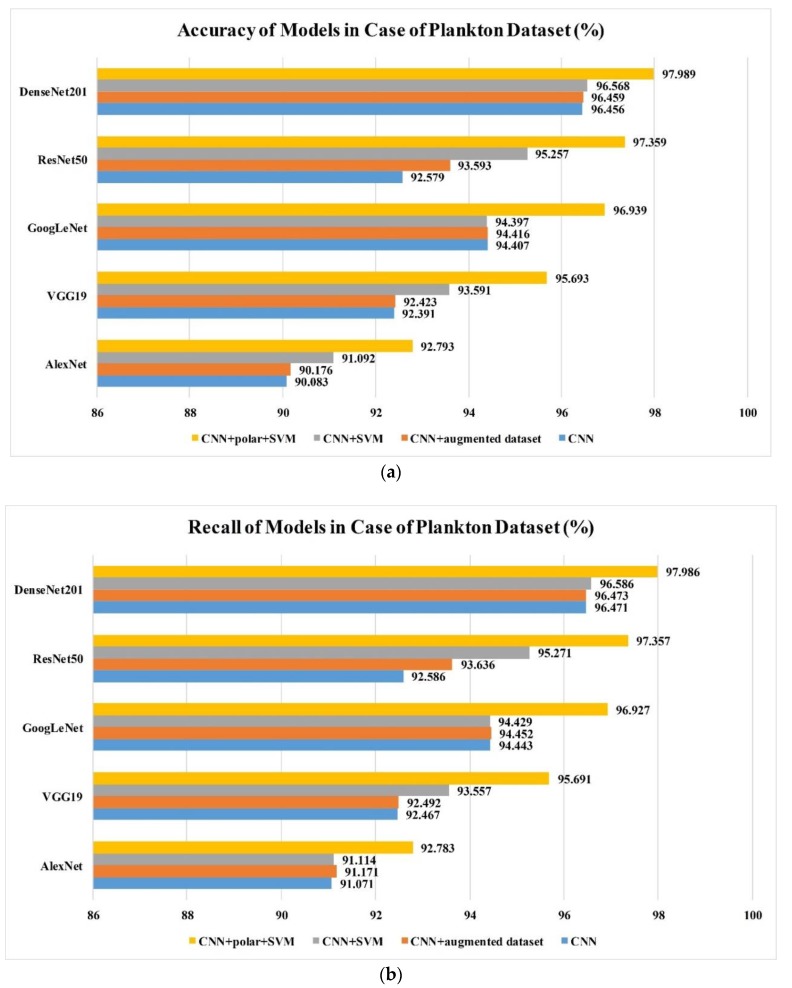

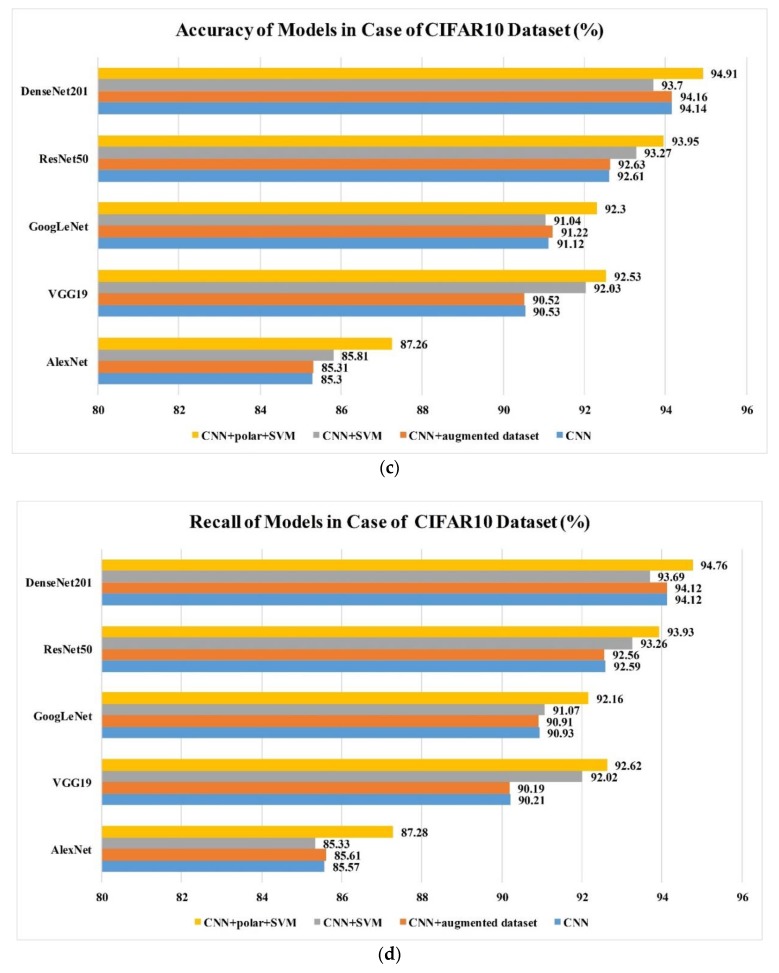
(**a**) Accuracy values of different models obtained using the proposed training method and plankton dataset; (**b**) recall rate values of different models obtained using the proposed training method and plankton dataset; (**c**) accuracy values of different models obtained using the proposed training method and CIFAR-10 dataset; (**d**) recall rate values of different models obtained using the proposed training method and CIFAR-10 dataset.

In this study, we analyzed the performance and the pertinent parameters of the four different classification methods against CIFAR-10 and the in situ plankton image datasets. The results are shown in Figure 9. These methods included a classical convolutional neural network (Method I); a classical convolutional neural network trained by the augmented dataset (Method II); a classical convolutional neural network with ordinary features combined with an SVM classifier (Method III); and a classical convolutional neural network with both ordinary and polar coordinate features combined with an SVM classifier (Method IIII). The feasibility of the proposed method is demonstrated by the results obtained on the public CIFAR-10 dataset in which Method IIII performed the best among all the methods. The classification accuracy and recall rate were the highest in Method IIII. Method III exhibited the next highest performance, followed by Method II and Method I. Moreover, it was observed that the classification accuracy and recall rate could be improved by partially replacing the totally connected layer of the convolutional neural network and the Softmax classification layer with the multiclass SVM model. Furthermore, the use of polar coordinates and the multiclass SVM classifier layer significantly improved the classification accuracy and recall rate. The improvement was in the range of approximately 2–5%, which was significant with respect to classification methods. The polar coordinate features allow the feature conversion of rotational characteristics into translational ones. Both the rotated and the existing ordinary features were then learned by the optimized convolutional neural network in order to increase the generalization ability of the proposed model. Compared with Method I, Method II uses an augmented dataset to enhance the generalization ability in rotation, however, the improvement is limited; even though the amount of data in the augmented dataset is 14 times that of the original, the training time will also increase greatly. For the proposed method, the improvement is much more significant; it demonstrates its effectiveness in features extraction and combination. In general, the same conclusion is applicable to both the open dataset (CIFAR10) and the plankton dataset, which shows its robustness to different datasets.

### 3.4. Discussion

**Figure 10:**
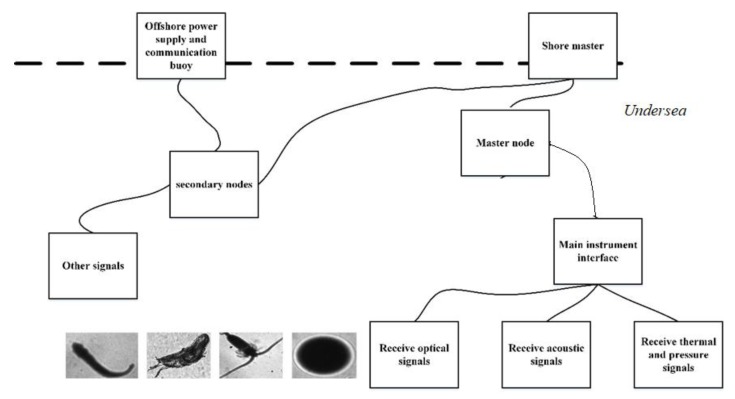
Diagram of undersea observation network.

The introduction of polar coordinate features allowed the conversion of the rotational features into translational features. As both the rotated features and the ordinary features were then learned by the convolutional neural network, the generalization ability of the model increased. Moreover, the model could be suitable for multi-pose and multi-angle images, which are very common in natural environments. The images in the plankton dataset were collected in the ocean and the camera angle was not fixed, so the creatures in the image have a variety of postures and angles. Consequently, in order to describe such images better, more rotated features are needed. Using polar coordinates combined with common features and rotation features, better results can be achieved when dealing with images without specific angles. Thus, the proposed method can be applicable for the undersea imager receiving optical signals of the tiny creatures in the undersea observation network (shown in Figure 10). It may also have better effect on the images taken by autopilot and with unmanned aerial vehicles (UAVs), and related work can be carried out in the future.

## 4. Summary and Outlook

We developed a training method for convolutional neural networks designed for the recognition and classification of plankton by applying the mechanism of the human eye or the polar coordinate system. When applying the classification and identification for in situ plankton images, the image was described in the polar coordinate system as well as the Cartesian coordinate system. This has enabled the construction of an optimized classification and recognition network available for in situ plankton images. This was constructed by exploiting the advantages of both coordinate systems and automatically adjusting the weights of two eigenvectors in network training. The model is trained, using 14,336 ROIs and tested, using 3,584 ROIs both from the in situ plankton images. The DenseNet201 + Polar + SVM model exhibited the highest classification accuracy (97.989%) and recall rate (97.986%) in the comparative tests. The accuracy of the proposed model was markedly higher than those of the initial classical convolutional neural networks when using the in situ plankton image data with the increase in classification accuracy and recall rate being 5.3% and 5.1% respectively. In addition, the proposed training method can improve the classification performance considerably when it is used on the public CIFAR-10 dataset, which consists of 10 categories with 50,000 training samples and 10,000 test samples. In this case, the DenseNet201 + Polar + SVM model showed the highest classification accuracy (94.91%) as well as the highest recall rate (94.76%).

One shortcoming of the proposed method is that it requires an extra neural network, specifically for the polar-transformed images; thus, it requires extra time in network training. However, as the structures of these two networks are similar, their hyper-parameters such as learning rate and their training epoch are the same. This will extract more features, and increases the accuracy as well as recall rate, and possesses a controllable efficient model. Another point to be noted is the resizing operation in the model. The images used as the network inputs were standardized to ensure that they had a uniform size. Thereafter, they are classified and recognized, using the convolutional neural network. This operation inevitably affected the morphological characteristics of the targets in the images. In future studies, a more optimal combination of the two different networks should be investigated to form a unified training and testing structure. Concretely, we will focus on extracting the two features with only one convolutional neural network in the future. In addition, the feature fusion part also needs to be completed with an SVM. We hope to simplify this point and put the feature fusion characteristic into the convolutional neural network by designing a specialized module. Finally, we will embed our proposed model into the undersea imager or UAV imager to achieve real-time image acquisition and classification.

## Figures and Tables

**Table 1:** Performances of different models with respect to the dataset constructed in the present study.

No.	Model (Detailed Structure)	Precision (%)	Recall Rate (%)	Time (ms/Sample)
1	AlexNet	90.083	91.071	5.601
2	VGG19	92.391	92.467	17.437
3	GoogLeNet	94.407	94.443	7.533
4	ResNet50	92.579	92.586	11.677
5	DenseNet201	96.456	96.471	23.281
6	AlexNet + augmented dataset	90.176	91.171	5.593
7	VGG19 + augmented dataset	92.423	92.492	17.451
8	GoogLeNet + augmented dataset	94.416	94.452	7.527
9	ResNet50 + augmented dataset	93.593	93.636	12.311
10	DenseNet201 + augmented dataset	96.459	96.473	23.297
11	AlexNet-fc + SVM	91.092	91.114	5.833
12	VGG19-fc + SVM	93.591	93.557	19.109
13	GoogLeNet-fc + SVM	94.397	94.429	7.630
14	ResNet50-fc + SVM	95.257	95.271	12.089
15	DenseNet201-fc + SVM	96.568	96.586	23.912
16	AlexNet-fc+AlexNet_Polar-fc + SVM	92.793	92.783	11.235
17	VGG19-fc+VGG19_Polar-fc + SVM	95.693	95.691	37.975
18	GoogLeNet-fc + GoogLeNet_Polar-fc + SVM	96.939	96.927	14.818
19	ResNet50-fc + ResNet50_Polar-fc + SVM	97.359	97.357	23.786
20	DenseNet201-fc + DenseNet201_Polar-fc + SVM	97.989	97.986	46.417

**Table 2:** Performances of different models with respect to the CIFAR-10 dataset.

No.	Model (Detailed Structure)	Precision (%)	Recall Rate (%)	Time (ms/Sample)
1	AlexNet	85.30	85.57	3.75
2	VGG19	90.53	90.21	16.29
3	GoogLeNet	91.12	90.93	5.48
4	ResNet50	92.61	92.59	9.10
5	DenseNet201	94.14	94.12	21.57
6	AlexNet + augmented dataset	85.31	85.61	3.71
7	VGG19 + augmented dataset	90.52	90.19	16.23
8	GoogLeNet + augmented dataset	91.22	90.91	5.51
9	ResNet50 + augmented dataset	92.63	92.56	9.12
10	DenseNet201 + augmented dataset	94.16	94.12	21.56
11	AlexNet-fc + SVM	85.81	85.33	4.32
12	VGG19-fc + SVM	92.03	92.02	17.05
13	GoogLeNet-fc + SVM	91.04	91.07	5.81
14	ResNet50-fc + SVM	93.27	93.26	10.35
15	DenseNet201-fc + SVM	93.70	93.69	21.63
16	AlexNet-fc + AlexNet_Polar-fc + SVM	87.26	87.28	8.83
17	VGG19-fc + VGG19_Polar-fc + SVM	92.53	92.62	36.32
18	GoogLeNet-fc + GoogLeNet_Polar-fc + SVM	92.30	92.16	10.90
19	ResNet50-fc + ResNet50_Polar-fc+SVM	93.95	93.93	19.72
20	DenseNet201-fc + DenseNet201_Polar-fc + SVM	94.91	94.76	43.95
